# The Impact of Adding Sugars to Milk and Fruit on Adiposity and Diet Quality in Children: A Cross-Sectional and Longitudinal Analysis of the Identification and Prevention of Dietary- and Lifestyle-Induced Health Effects in Children and Infants (IDEFICS) Study

**DOI:** 10.3390/nu10101350

**Published:** 2018-09-21

**Authors:** Marika Dello Russo, Wolfgang Ahrens, Stefaan De Henauw, Gabriele Eiben, Antje Hebestreit, Yannis Kourides, Lauren Lissner, Denes Molnar, Luis A. Moreno, Valeria Pala, Toomas Veidebaum, Alfonso Siani, Paola Russo, on behalf of the IDEFICS Consortium

**Affiliations:** 1 Institute of Food Sciences, CNR, 83100 Avellino, Italy; marika.dellorusso@isa.cnr.it (M.D.R.); prusso@isa.cnr.it (P.R.); 2 Leibniz Institute for Prevention Research and Epidemiology—BIPS, 28359 Bremen, Germany; ahrens@leibniz-bips.de (W.A.); hebestr@leibniz-bips.de (A.H.); 3 Institute of Statistics, University of Bremen, 28359 Bremen, Germany; 4 Department of Public Health, Faculty of Medicine and Health Sciences, Ghent University, 9000 Ghent, Belgium; stefaan.dehenauw@ugent.be; 5 Department of Biomedicine and Public Health, School of Health and Education, University of Skövde, 54128 Skövde, Sweden; gabriele.eiben@his.se; 6 Research and Education Institute of Child Health, 2040 Strovolos, Cyprus; kourides@cytanet.com.cy; 7 Section for Epidemiology and Social Medicine, Sahlgrenska Academy, University of Gothenburg, 40530 Gothenburg, Sweden; lauren.lissner@gu.se; 8 Department of Paediatrics, University of Pécs, 7624 Pécs, Hungary; molnar.denes@pte.hu; 9 GENUD (Growth, Exercise, Nutrition and Development) Research Group, University of Zaragoza, 50009 Zaragoza, Spain; lmoreno@unizar.es; 10 Epidemiology and Prevention Unit, Fondazione IRCSS Istituto Nazionale dei Tumori, 20133 Milan, Italy; Valeria.Pala@istitutotumori.mi.it; 11 National Institute for Health Development, 11619 Tallinn, Estonia; toomas.veidebaum@tai.ee

**Keywords:** added sugars, milk, fruit, children, obesity, cohort study, healthy diet score, dietary pattern

## Abstract

Sugar, particularly as free sugars or sugar-sweetened beverages, significantly contributes to total energy intake, and, possibly, to increased body weight. Excessive consumption may be considered as a proxy of poor diet quality. However, no previous studies evaluated the association between the habit of adding sugars to “healthy” foods, such as plain milk and fresh fruit, and indicators of adiposity and/or dietary quality in children. To answer to these research questions, we Panalysed the European cohort of children participating in the IDEFICS study. Anthropometric variables, frequency of consumption of sugars added to milk and fruit (SAMF), and scores of adherence to healthy dietary pattern (HDAS) were assessed at baseline in 9829 children stratified according to age and sex. From this cohort, 6929 children were investigated again after two years follow-up. At baseline, a direct association between SAMF categories and adiposity indexes was observed only in children aged 6–<10 years, while the lower frequency of SAMF consumption was significantly associated with a higher HDAS. At the two year follow-up, children with higher baseline SAMF consumption showed significantly higher increases in all the anthropometric variables measured, with the exception of girls 6–<10 years old. The inverse association between SAMF categories and HDAS was still present at the two years follow-up in all age and sex groups. Our results suggest that the habit to adding sugars to foods that are commonly perceived as healthy may impact the adherence to healthy dietary guidelines and increase in adiposity risk as well.

## 1. Introduction

The childhood obesity pandemic being currently observed in most developed and developing countries urges the identification of effective strategies for its prevention and treatment [1]. One key modifiable factor for obesity prevention is energy intake, and its reduction could be achieved through nutritional and behavioural interventions [1]. Sugar, particularly as free sugar or sugar-sweetened beverages (SSB), significantly contributes to total energy intake, and, possibly, to increased body weight [2]. Excessive sugars consumption may be also considered as a proxy of poor diet quality [3,4].

During the last decades, the prevalence of overweight/obesity increased, along with the consumption of sugars, suggesting an association between sugar consumption and obesity [5], although the causal importance of this association has been questioned [6]. A trend toward decreasing sugar consumption has been observed worldwide [7], and recently confirmed for adolescents and young adults [8]. However, recent data on the European children participating to the Identification and Prevention of Dietary-and Lifestyle-Induced Health Effects in Children and Infants (IDEFICS) study indicate that the mean total intake is still high [9], particularly in the light of the WHO Guidelines for sugars intake released in 2015 [3]. The evidence about sugar intake in European countries has been recently reviewed by Azaïs-Braesco et al. [10], confirming the high intake of total and added sugars in Europe, especially in children, and identifying sweet products and beverages as the major contributors to added sugar intakes. Of note, the authors highlighted the many limitations in the interpretation of the available data, which is mainly due to important items, such as dietary data collection, food composition tables, or estimation of added sugars [10], not considering the varying definition of sugars used in different context [11].

Actually, international bodies generally agree on the term “sugars” to cover monosaccharides and disaccharides present in or added to foods, although some differences exist regarding the terms “added sugars” or “free sugars” [3,12,13].

Dietary guidances, differentiating free sugars from sugars that are naturally contained in foods, such as fruit and milk, recommend to reduce the intake of free sugars, replacing their energy contribution with starches, sugars contained within the cellular structure of foods and in milk and milk products [13]. As a matter of fact, daily consumption of non-fat (skim) or low-fat milk and dairy products, and fruit is widely recognized as part of the healthy diet for children and adolescents [14].

However, the effectiveness of the recommendations to increase the consumption of these “healthy” foods may be reduced if sugars are added to them, as often happens, particularly during childhood. The innate desire and preference of children for sweet foods [15] may indeed lead the parents or caregivers to add sugar to milk and fruit to favour the consumption of such foods, perceived as “healthy”, underestimating the caloric burden that is associated with these added sugars [16].

Various foods and food components have been considered to play a positive or negative role in the development of obesity in childhood and adolescence. However, it is plausible that, with the exception of SSB, whose association with obesity has been consistently reported [17], overall dietary patterns may better explain obesity risk than individual food components [18].

Of note, consumption of SSB appears to be associated to, and may be even considered, a proxy of an overall unhealthy dietary pattern [19,20].

As a consequence, the evaluation of added sugar intake was focused particularly on SSB, along with sweet snacks as major sources, overlooking the possible effect of the consumption of “healthy” foods to whom sugar is added. Several studies investigated the association of the consumption of ready-to-drink flavoured milk beverages with energy intake and obesity, as recently reviewed by Fayet-Moore [21] and Patel et al. [22]. To our knowledge, there are no previous studies evaluating the association between the habit of adding sugars to “healthy” foods, such as plain milk and fresh fruit, and indicators of adiposity and/or dietary quality.

Therefore, the novel research question of this study is to investigate in the large European cohort of children participating to the IDEFICS survey, both cross-sectionally and prospectively, whether the habit to add sugar to milk and/or fruit (sugars added to milk and/or fruit, SAMF) is associated to adiposity indexes and/or to the quality of the diet, as assessed by a healthy diet score (Healthy Dietary Adherence Score, HDAS) [23].

## 2. Subjects and Methods

### 2.1. Study Population

The IDEFICS study (Identification and prevention of dietary-and lifestyle-induced health effects in children and infants), registration number ISRCTN62310987, investigated the aetiology of diet- and lifestyle-related diseases and disorders with a strong focus on overweight and obesity in a large population-based cohort of 16,228 children aged 2–<10 years, who were recruited through schools and kindergartens in eight European countries (Belgium, Cyprus, Estonia, Germany, Hungary, Italy, Spain, and Sweden). Since the IDEFICS study included an intervention program, in each country two comparable areas (intervention and control region) were selected [24]. Details of the general design, instruments, and survey characteristics can be found elsewhere [25]. The baseline examination was carried out from September 2007 to May 2008 and the follow-up examination two years later, between September 2009 and May 2010.

**Figure 1:**
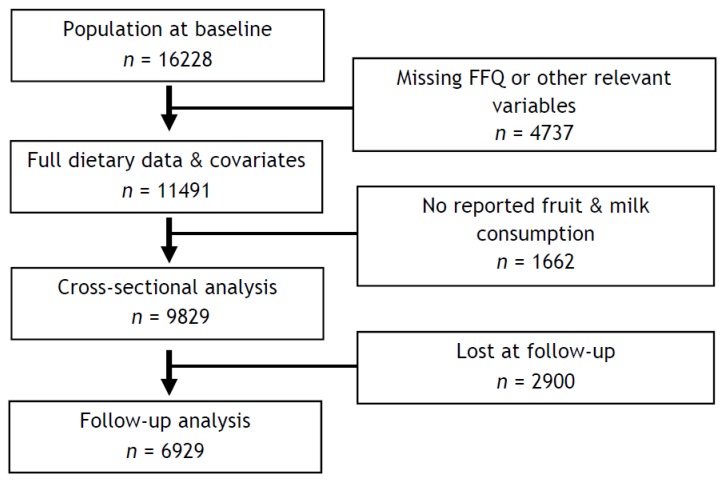
Participant flow chart.

From the full survey sample of 16,228 children, a total of 11,491 children (70.8% of the sample), was eligible for the cross-sectional analysis, after the exclusion of participants (*n* = 4737) for whom specific variables were missing (food frequency questionnaire and physical activity of the child, family income). Further 1662 children were excluded due to not reported consumption of milk and/or fruit. The final analysis was conducted on 9829 children that were stratified according to their age at baseline and sex (2–<6 years: boys 2368, girls 2228; 6–<10 years: boys 2579, girls 2654). Excluded children were not different from those that were included in the analysis with regard to the variables of interest (data not shown). Two years after baseline, the children participated in the follow-up examination, and all the examinations were repeated with comparable procedures. For the prospective analysis, a total of 6929 children were examined a second time two years later, during the follow-up survey. The participants flow chart is reported in Figure 1.

The study was conducted according to the standards of the Declaration of Helsinki and approved by local Ethics Committees of each participating centre (1. Belgium: Ethics Committee of the Gent University Hospital, 15/10/2007, ref: No. EC UZG 2007/243 and 19/02/2013, No. B670201316342. 2. Cyprus: Cyprus National Bioethics Committee, 12/07/2007, ref: No. EEBK/EM/2007/16 and 21/Feb/2013, No. EEBK/ETI/2012/33. 3. Estonia: Tallinn Medical Research Ethics Committee (TMREC), 14/06/2007, ref: No. 1093 and 17/January 2013, No. 128. 4. Germany: Ethic Commission of the University of Bremen, 16/01/2007 and 11/12/2012. 5. Hungary: Medical Research Council, 21/Jun/2007, ref: 22-156/2007-1018EKU and 18/12/2012, 4536/2013/EKU. 6. Italy: Ethics Committee of the Local Health Authority (ASL) in Avellino, 19/06/2007, ref: No. 2/CE and 18/Sep/2012, No. 12/12. 7. Spain: Ethics Committee for Clinical Research of Aragon (CEICA), 20/06/2007, ref: No. PI07/13 and 13/Feb/2013, No. PI13/0012. 8. Sweden: Regional Ethics Research Board in Gothenburg, 30/07/2007, ref: No. 264-07 and 10/Jan/2013, No. 927-12). Parents were asked to sign a written informed consent, whereas children provided their oral assent.

### 2.2. Physical Examination

The examination programme included standard anthropometric measures, blood pressure measurements and blood samples collection. We describe below the measurements that were considered in the present analysis. Body weight, height, waist circumference, and skinfold thickness were measured. A detailed description of the anthropometric measurements in the IDEFICS study, including intra- and inter-observer reliability, has been published by Stomfai et al. [26]. Weight was determined to the nearest 0.1 kg using an electronic scale (Tanita BC 420 SMA, Tanita Europe GmbH, Sindelfingen, Germany) with children that were wearing only light clothes without shoes. Height was measured while using a calibrated stadiometer instrument (Seca 225, Seca GmbH & Co., KG., Hamburg, Germany) with approximation of 0.1 cm. BMI was calculated as weight (in kg) divided by height squared (in m^2^). Sex- and age-specific BMI *z*-scores were calculated based on Cole & Lobstein [27].

Waist circumference (WC) was measured while using an inelastic tape (Seca 200, Seca GmbH & Co., KG., Hamburg, Germany), range 0 ± 150 cm, at the midpoint between the iliac crest and the lower border of tenth rib with the subject in a standing position with arms being relaxed on the sides and recorded at the nearest 0.1 cm. Skinfold thickness (mm) was measured twice on the right side of the body to the nearest 0.2 mm with a skinfold calliper (Holtain, range 0–40 mm, Holtain Ltd., Pembrokeshire, UK), as described in Nagy et al. [28]. The sum of subscapular (mm) and triceps (mm) skinfold thickness was used for the calculation of body fat mass (BFM), according to Slaughter [29].

### 2.3. Parental Questionnaire

Parents were asked to fill in the questionnaire for their children (date of birth, physical activity and lifestyle factors, and personal and familial medical history) and for themselves (age, self-reported weight and height, educational level, and occupation and family income). Income levels were grouped into four categories (low, medium, medium-high, high). As for physical activity, parents were invited to indicate how many minutes per day their children used to spend in outdoor activities (i.e., time spent playing in the yard or outdoor recreation area as swimming pool, zoo, and/or park) both on weekdays and weekend days. An additional question investigated whether or not the child would have attended a club or sport association to exercise.

### 2.4. Dietary Assessment

The food frequency consumption was evaluated by means of a specific section of the Children’s Eating Habits Questionnaire (CEHQ-FFQ), a reproducible screening instrument [30,31,32,33]. A pilot study found the CEHQ-FFQ to be reproducible with mean Kappa coefficients ranging from 0.41 to 0.60 and Spearman’s correlation higher than 0.5% for 81% of the food items [30]. Further, a validation study against repeated 24-h dietary recall found that under 12% of the food groups were classified in the wrong quartile of intake [32]. The validity and reliability of the IDEFICS CEHQ-FFQ with regard to the frequencies of milk consumption were further confirmed by a urinary biomarker-based analysis on the IDEFICS population [31].

CEHQ-FFQ asked for the consumption frequency of 43 pan-European food items of 14 food groups, which were designed to identify specific dietary patterns shown to be related to overweight or obesity in children. The sequence of food items queried for each food group was planned to avoid confusion and to minimise the risk of double reporting for the same food. CEHQ-FFQ was developed in English and then translated into local language with the aim to obtain comparable data on eating behaviour across all the participating centres. Parents were asked to complete the food questionnaire answering how many times their child had consumed food and drink they knew about, thus in their presence or outside the school canteen or childcare meal provision setting, referring to a typical week of the previous month. Frequencies of consumption were classified into eight categories: “never/less than once a week”, “1–3 times per week”, “4–6 times per week”, “once per day”, “twice per day”, “three times per day”, “four or more times per day”, and “I have no idea”.

The habit of adding sugar to milk and fruit was evaluated selecting the item “Sweetened milk (e.g., addition of sugar, chocolate powder, honey, etc.)” and the item “Fresh fruits (also as freshly squeezed, fruit smoothie) with added sugar” of the CEHQ-FFQ. These two items were combined into a new variable named “added sugars”. Combined frequencies of consumption of sugars added to milk and/or fruit (SAMF) were differentiated into three categories: “rarely” (that contains the category “never/less than once a week”), “weekly” (when the consumption is less than one time per day) and “daily” (when the added sugar consumption is once or more times per day). Fruit and milk consumption was calculated by summing all the items in CEHQ-FFQ regarding these foods: “Plain unsweetened milk”, “Sweetened milk (e.g., addition of sugar, chocolate powder, honey, etc.)”, “Fresh fruits (also freshly squeezed, fruit smoothie) without added sugar”, “Fresh fruits (also freshly squeezed, fruit smoothie) with added sugar”.

To assess children´s diet quality, a Healthy Dietary Adherence Score (HDAS) was calculated from CEHQ-FFQ as a measure of the degree to which children’s dietary intake follows nutrition guidelines [23]. The HDAS was developed according to the principles reviewed by Waijers et al. [34]. Briefly, healthy dietary recommendations include: limit the intake of refined sugars, reduce fat intake, especially of saturated fat, choose whole meal when possible, consume 400–500 g of fruits and vegetables per day and fish 2–3 times per week. Hence, the HDAS contains five components: sugar, fat, whole meal, fruits and vegetables, and fish. Each component has a minimum score of 0 and a maximum score of 10, summed to a maximum score of 50, where the highest score indicates the highest possible adherence to the dietary guidelines. A more detailed description of the HDAS can be found in Ardvisson et al. [23].

### 2.5. Statistical Analysis

All of the analyses were performed separately in boys and girls and stratified by baseline age groups (2–<6 years, ≥6–<10 years). Data were expressed as mean and standard deviation (SD) or 95% confidence intervals (95% CI), as indicated in the tables. The cross-sectional analysis of the categories of SAMF with anthropometric variables and HDAS was performed using analysis of co-variance (GLM, General Linear Model). The models were adjusted by age, country, family income, physical activity, and fruit and milk consumption of the child (obtained by the sum of the frequency of consumption of unsweetened and sweetened milk and fruit). *p* values for linear trend were calculated. Longitudinal analyses were performed using the two years variation in the adiposity variables (follow-up value minus baseline value) and HDAS at follow-up across the baseline categories of SAMF. Models were adjusted by the respective baseline value of the outcome variable of interest and by age, country, family income, physical activity, intervention/control study group, and fruit and milk consumption of the child (obtained by the sum of the frequency of consumption of unsweetened and sweetened milk and fruit). *p* values for linear trend were calculated. A two-tailed *p* value less than 0.05 was considered as statistically significant. Statistical analyses were performed while using IBM SPSS Statistics (Version 23.0. IBM Corp., Armonk, NY, USA)

## 3. Results

The characteristics of the population at baseline, and at the end of the two-years follow-up are reported in Table 1.

### 3.1. Cross-Sectional Analysis

Mean adiposity measures and the HDAS stratified by baseline categories of SAMF, after adjustment for covariates, are presented in Table 2.

At multiple regression analysis, in younger boys and girls, no association was evident between SAMF and adiposity indexes, while SAMF consumption was significantly and inversely associated with HDAS, in both boys and girls of all age groups. In older boys and girls, a positive association was observed between SAMF categories and adiposity indexes, with increasing BMI *z*-score values, sum of skinfolds, and percentage of body fat across SAMF categories.

### 3.2. Prospective Analysis

Table 3 reports the changes in anthropometric variables in boys and girls over the two years follow-up across the SAMF categories defined at baseline.

Younger children with higher baseline SAMF consumption showed significantly higher increases in all the anthropometric variables measured. In older children, some differences between boys and girls became apparent. While, in boys, all anthropometric parameters were positively associated with SAMF consumption, in girls only a positive association with WC *z*-score was observed. The significant association between SAMF categories and HDAS was still present at the two years follow-up in all age and sex groups.

## 4. Discussion

A consistent body of evidence recognized the contribution of added sugars to poor quality, energy dense diet, and, possibly, to the development of conditions such as obesity and increased cardiometabolic risk in children and adolescents [35]. In 2015, the WHO released a strong recommendation to reduce the intake of free sugars to less than 10% of total energy intake in both adults and children, and a conditional recommendation to further reduce their intake to less than 5% of total energy intake [3]. Although the terminology that is used to describe dietary sugars is still under debate [11] and its discussion is beyond the scope of this paper, there is general agreement that naturally occurring sugars in dairy foods and in intact (fresh, cooked, or dried) fruit and vegetables are excluded by the common definition of added sugar, and might have different impact on health outcomes [11,36]. Actually, milk (particularly low-fat milk) and fruit are considered to be foods with a favourable nutrient profile and are consistently recognized as part of the healthy diet for children and adolescents [14]. However, to the best of our knowledge, the impact of adding sugars to these healthy foods on health outcomes, such as adiposity indexes, or on the adherence to healthy dietary guidelines, was not yet investigated.

Cross-sectional analyses revealed that the higher frequency of SAMF consumption was significantly associated with higher adiposity indexes in boys and girls in the age range 6–10 years. When examining the younger children group (2–<6 years), no evidence of such an association was observed. Since this is the first observation of the impact of SAMF on adiposity indexes, we do not have a plausible explanation for the discrepancy observed in younger and older children with regard to this association. However, based on the large body of evidence showing that the liking for sweet taste is innate, and that children show higher levels of sweet preferences than adults [37], we can speculate that, during the first years of life, the appetite for sweet may be a protective mechanism that is driven by the child’s need for calories during growth [38,39]. Thus, the higher sugar intake during early development did not show negative effects than compared to later in the life. However, further studies are needed to explore whether there is an age-related modulation of the association between SAMF consumption and adiposity in children.

Interestingly, a significant and linear inverse association was observed between SAMF and our score of adherence to healthy dietary guidelines, indicating that the adoption of a habit to add sugars to healthy foods can be a trigger for longer term inflation of dietary quality. Our longitudinal analyses showed that a higher frequency of SAMF consumption at baseline predicted a higher increase of all anthropometric indexes over the two-years follow up in all children groups with the exception of older girls, in which only a positive trend for waist circumference was observed. The observation that HDAS was inversely associated with the categories of SAMF consumption consistently remained even after follow-up. In summary, our results suggest that the habit of adding sugars to milk and fruit may have a not negligible impact on adiposity indexes, and on adherence to healthy dietary guidelines. Our results are in agreement with the conclusion of the recent systematic review by Patel et al. [22], suggesting that while the consumption of flavoured milk might promote overall milk intake, its potential adverse effects on caloric intake and possibly obesity for children and adolescents need further investigation.

### Strenghts & Limitations

Our study has several strengths. First, this is, to our knowledge, the first study that examined the association between the frequency of consumption of SAMF and adiposity measures in children. Our findings add to the current literature in that we were able to examine the association between SAMF and adherence to healthy dietary guidelines. We used the *a priori* diet score, HDAS, as calculated from the FFQ used in the IDEFICS survey [23], as an indicator of the overall quality of the diet. Of note, the HDAS was based on healthy dietary guidelines common for the eight European countries that were included in the IDEFICS study, hence substantial evidence of beneficial health effects are underlying the foods and beverages included [23]. Other important strengths of the study are its multicentric nature, the large sample size, and the longitudinal design. We used standardized phenotypic measurements within the eight European countries participating in the survey. All of the measurements were conducted according to detailed standard operation procedures. In addition, subsamples of study subjects were repeatedly examined to calculate the inter- and intra-observer reliability of anthropometric measurements [26].

Despite the many strengths, there are also limitations. The present analysis, based on the CEHQ-FFQ, does not allow for a quantitative estimate of intakes, thus incurring in possible over- or under-estimation biases. However, this instrument has been found to be reproducible [30,31,32]. To account for the putative protective effect of milk and fruit consumption on adiposity [40,41], which might have confounded the observed associations, all of the analyses were adjusted for the frequency of consumption of milk and fruit at baseline. 

## 5. Conclusions

Given the innate preference of children for sweet [37], it is conceivable that parents might be inclined to make milk and fruit sweeter by adding variable amount of sugar to these foods to favour their consumption, considered as healthy according to dietary recommendations. Although there is some evidence in favour of the addition of small amounts of sugar to encourage the intake of nutrients-rich foods, such as fruit and milk [42], our results suggest that leaving to discretionary parenting practices the use (or abuse) of adding sugars to increase the acceptability of such foods may, in the longer run, be counterproductive and result in deviations from the adherence to healthy dietary guidelines, and hence also an increase in adiposity risk.

## Figures and Tables

**Table 1:** Characteristics of the population at baseline and at the two-years follow-up.

Children 2–<6 Years Old						
	Baseline (all)		Baseline (subgroup *)		Follow-up (2 years)	
	Boys (*n* = 2368)	Girls (*n* = 2228)	Boys (*n* = 1648)	Girls (*n* = 1556)	Boys (*n* = 1648)	Girls (*n* = 1556)
Age (years)	4.2 ± 0.9	4.2 ± 0.9	4.2 ± 0.9	4.2 ± 0.9	6.2 ± 1.0	6.2 ± 0.9
BMI *z*-score	0.08 ± 1.13	0.12 ± 1.10	0.08 ± 1.08	0.12 ± 1.11	0.20 ± 1.17	0.26 ± 1.12
WC *z*-score	−0.13 ± 1.20	−0.32 ± 1.33	−0.12 ± 1.16	−0.29 ± 1.34	0.27 ± 1.23	0.13 ± 1.32
SS, mm	26.8 ± 8.5	30.5 ± 10.0	26.5 ± 8.0	30.7 ± 9.9	16.5 ± 6.8	19.1 ± 7.2
Body fat, %	31.0 ± 6.0	38.4 ± 6.6	30.9 ± 6.0	38.5 ± 6.5	26.1 ± 6.2	32.5 ± 6.1
HDAS	23.3 ± 9.0	23.4 ± 9.0	23.5 ± 8.9	23.9 ± 9.1	23.7 ± 9.2	24.5 ± 9.2
**Children 6–<10 Years Old**						
	Baseline (all)		Baseline (subgroup)		Follow-up (2 years)	
	Boys (*n* = 2579)	Girls (*n* = 2654)	Boys (*n* = 1834)	Girls (*n* = 1891)	Boys (*n* = 1834)	Girls (*n* = 1891)
Age (years)	7.5 ± 0.8	7.5 ± 0.8	7.4 ± 0.8	7.4 ± 0.8	9.4 ± 0.8	9.4 ± 0.8
BMI *z*-score	0.45 ± 1.20	0.48 ± 1.14	0.43 ± 1.19	0.44 ± 1.11	0.52 ± 1.19	0.48 ± 1.10
WC *z*-score	0.61 ± 1.43	0.41 ± 1.48	0.61 ± 1.32	0.38 ± 1.47	0.78 ± 1.23	0.59 ± 1.28
SS, mm	33.4 ± 18.4	38.6 ± 19.3	33.1 ± 17.8	37.8 ± 18.5	21.6 ± 11.3	24.5 ± 11.1
Body fat, %	26.3 ± 7.2	31.8 ± 6.8	26.1 ± 7.0	31.5 ± 6.7	28.1 ± 8.3	32.7 ± 7.5
HDAS	21.5 ± 8.6	22.1 ± 8.9	21.9 ± 8.7	22.6 ± 9.0	22.4 ± 8.7	23.1 ± 9.0

BMI, body mass index; WC, waist circumference; SS, Sum of Skinfolds, HDAS, Healthy Dietary Adherence Score. Values are mean ± SD, * Children who participated to the 2 years follow-up.

**Table 2:** Anthropometric variables and Healthy Dietary Adherence Score (HDAS) at baseline across sugars added to milk and/or fruit (SAMF) categories

Children 2–<6 Years Old								
	Boys				Girls			
SAMF categories	Rarely (*n* = 670)	Weekly (*n* = 698)	Daily (*n* = 1000)	*p* for trend	Rarely (*n* = 645)	Weekly (*n* = 704)	Daily (*n* = 879)	*p* for trend
BMI *z*-score	0.13 (0.04; 0.21)	−0.04 (−0.13; 0.04)	0.14 (0.07; 0.21)	ns	0.16 (0.07; 0.24)	0.06 (−0.02; 0.14)	0.15 (0.08; 0.23)	ns
WC *z*-score	−0.17 (−0.27; −0.07)	−0.14 (−0.24; 0.04)	−0.09 (−0.17; −0.01)	ns	−0.29 (−0.40; −0.18)	−0.32 (−0.43; −0.22)	−0.33 (−0.42; −0.23)	ns
SS, mm *	26.7 (25.8; 27.6)	26.5 (25.7; 27.4)	26.9 (26.3; 27.6)	ns	30.3 (29.2; 31.4)	29.9 (28.9; 30.8)	31.0 (30.2; 31.8)	ns
Body fat, % *	31.3 (30.9; 31.7)	30.3 (30.0; 30.7)	31.3 (31.0; 31.6)	ns	38.5 (38.1; 38.9)	38.1 (37.7; 38.5)	38.7 (38.3; 39.1)	ns
HDAS *	27.9 (27.2; 28.5)	23.9 (23.3; 24.5)	19.7 (19.2; 20.2)	<0.0001	27.5 (26.9; 28.1)	24.1 (23.5; 24.7)	19.8 (19.3; 20.4)	<0.0001
**Children 6–<10 Years Old**								
	Boys				Girls			
SAMF categories	Rarely (*n* = 630)	Weekly (*n* = 817)	Daily (*n* = 1132)	*p* for trend	Rarely (*n* = 653)	Weekly (*n* = 913)	Daily (*n* = 1088)	*p* for trend
BMI *z*-score	0.40 (0.31; 0.49)	0.38 (0.30; 0.46)	0.53 (0.46; 0.60)	0.02	0.39 (0.31; 0.48	0.43 (0.35; 0.50)	0.57 (0.50; 0.64)	0.002
WC *z*-score	0.58 (0.47; 0.70)	0.52 (0.42; 0.62)	0.70 (0.61; 0.78)	ns	0.36 (0.25; 0.47)	0.36 (0.27; 0.46)	0.48 (0.39; 0.57)	ns
SS, mm *	31.3 (29.4; 33.1)	32.4 (30.9; 33.9)	34.9 (33.7; 36.0)	0.001	37.3 (36.4; 39.2)	36.5 (34.9; 38.0)	40.6 (39.3; 41.9)	0.001
Body fat, % *	25.8 (25.2; 26.3)	25.7 (25.2; 26.2)	27.0 (26.6; 27.5)	<0.0001	31.4 (30.9; 32.0)	31.3 (30.9; 31.8)	32.5 (32.0; 32.9)	0.002
HDAS *	25.9 (25.3; 26.5)	22.1 (21.6; 22.7)	18.6 (18.2; 19.1)	<0.0001	26.2 (25.6; 26.8)	23.0 (22.5; 23.6)	19.0 (18.5; 19.5)	<0.0001

BMI, body mass index; WC, waist circumference; SS, Sum of Skinfolds; HDAS, Healthy Dietary Adherence Score; SAMF, sugar added to milk and fruit; ns, not significant. Values are mean (95% CI). Analysis adjusted for country, control or intervention region, physical activity, family income and fruit&milk consumption. * Adjusted for the same variables as above plus age.

**Table 3:** Changes in anthropometric variables and HDAS over the two-year follow-up across the SAMF defined at baseline.

Children 2–<6 Years Old at Baseline								
	Boys				Girls			
SAMF categories	Rarely (*n* = 490)	Weekly (*n* = 488)	Daily (*n* = 670)	*p* for trend	Rarely (*n* = 469)	Weekly (*n* = 477)	Daily (*n* = 610)	*p* for trend
BMI *z*-score	0.06 (−0.01; 0.13)	0.10 (0.03; 0.16)	0.19 (0.13; 0.25)	0.005	0.10 (0.04; 0.16)	0.10 (0.04; 0.16)	0.19 (0.14; 0.24)	0.03
WC *z*-score	0.37 (0.29; 0.45)	0.41 (0.34; 0.49)	0.55 (0.49; 0.62)	0.001	0.42 (0.33; 0.50)	0.41 (0.33; 0.50)	0.56 (0.49; 0.64)	0.01
SS *	−10.1 (−10.6; −9.5)	−10.2 (−10.7; −9.7)	−9.4 (−9.8; −9.0)	0.05	−11.7 (−12.2; −11.1)	−11.3 (−11.9; −10.8)	−10.8 (−11.2; −10.3)	0.02
Body fat *	−5.1 (−5.5; −4.6)	−5.0 (−5.4; −4.6)	−4.3 (−4.7; −4.0)	0.009	−6.0 (−6.4; −5.6)	−6.3 (−6.7; −5.9)	−6.0 (−6.4; −5.7)	ns
HDAS * at follow-up	27.7 (26.9; 28.5)	24.4 (23.6; 25.2)	20.1 (19.5; 20.9)	<0.0001	27.8 (27.0; 28.6)	25.2 (24.4; 26.0)	21.2 (20.5; 22.0)	<0.0001
**Children 6–<10 Years Old at Baseline**								
	Boys				Girls			
SAMF categories	Rarely (*n* = 466)	Weekly (*n* = 559)	Daily (*n* = 809)	*p* for trend	Rarely (*n* = 462)	Weekly (*n* = 643)	Daily (*n* = 786)	*p* for trend
BMI *z*-score	0.01 (−0.05; 0.06)	0.07 (0.03; 0.12)	0.15 (0.10; 0.19)	<0.0001	0.06 (0.02; 0.10)	0.01 (−0.03; 0.04)	0.07 (0.04; 0.10)	ns
WC *z*-score	0.11 (0.05; 0.17)	0.16 (0.11; 0.22)	0.19 (0.14; 0.24)	0.05	0.16 (0.08; 0.23)	0.18 (0.12; 0.25)	0.25 (0.19; 0.31)	0.05
SS *	−11.1 (−11.8; −10.3)	−10.7 (−11.2; −10.1)	−10.3 (−10.7; −9.8)	0.05	−12.8 (−13.4; −12.1)	−13.2 (−13.8; −12.6)	−12.6 (−13.0; −12.2)	ns
Body fat *	1.5 (1.1; 1.9)	2.0 (1.7; 2.4)	2.4 (2.1; 2.7)	0.001	1.2 (0.9; 1.5)	1.0 (0.7; 1.2)	1.4 (1.1; 1.7)	ns
HDAS * at follow-up	25.8 (25.0; 26.6)	23.0 (22.2; 23.7)	20.1 (19.5; 20.7)	<0.0001	26.4 (25.5; 27.2)	24.3 (23.6; 25.0)	20.1 (19.5; 20.7)	<0.0001

Abbreviations: BMI, body mass index; WC, waist circumference; SS, Sum of Skinfolds; HDAS, Healthy Dietary Adherence Score; SAMF, sugar added to milk and fruit; ns, not significant. Changes calculated as (“value” at follow-up—“value” at baseline). Values are mean (95% CI). Analysis adjusted for country, intervention/control study group, physical activity, family income, fruit and milk consumption, and baseline correspondent value for the delta variables. * Adjusted for the same variables as above plus age.
